# l-Alanine methyl ester hydro­chloride monohydrate

**DOI:** 10.1107/S160053681100420X

**Published:** 2011-02-09

**Authors:** Martin Lutz, Arie Schouten

**Affiliations:** a Bijvoet Center for Biomolecular Research, Crystal and Structural Chemistry, Faculty of Science, Utrecht University, Padualaan 8, 3584 CH Utrecht, The Netherlands

## Abstract

The enanti­opure title compound, C_4_H_10_NO_2_^+^·Cl^−^·H_2_O, forms a two-dimensional network by inter­molecular hydrogen bonding parallel to (010). Non-merohedral twinning with a twofold rotation about the reciprocal c* axis as twin operation was taken into account during intensity integration and structure refinement. This twinning leads to alternative orientations of the stacked hydrogen-bonded layers.

## Related literature

For the related l-serine methyl ester hydro­chloride, see: Schouten & Lutz (2009 ▶). For the theory of twin formation, see: Cahn (1954 ▶). Twin integration is based on Schreurs *et al.* (2010 ▶) and the twin refinement on Herbst-Irmer & Sheldrick (2002 ▶). The methods of Flack (1983 ▶) and Hooft *et al.* (2008 ▶) were used for the absolute structure determination.
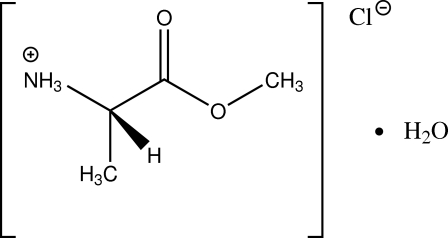


## Experimental

### 

#### Crystal data

C_4_H_10_NO_2_^+^·Cl^−^·H_2_O
*M* *_r_* = 157.60
Triclinic, 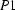

*a* = 4.9461 (4) Å
*b* = 6.0134 (4) Å
*c* = 6.6853 (5) Å
α = 101.833 (4)°
β = 93.533 (3)°
γ = 92.112 (4)°
*V* = 194.00 (2) Å^3^
*Z* = 1
Mo *K*α radiation
μ = 0.44 mm^−1^
*T* = 110 K
0.39 × 0.29 × 0.12 mm

#### Data collection

Nonius KappaCCD diffractometer
Absorption correction: multi-scan (*TWINABS-2008/4*; Sheldrick, 2008*a* ▶) *T*_min_ = 0.69, *T*_max_ = 0.75
11388 measured reflections
3213 independent reflections
3180 reflections with *I* > 2σ(*I*)
*R*_int_ = 0.019

#### Refinement

*R*[*F* ^2^ > 2σ(*F* ^2^)] = 0.015
*wR*(*F* ^2^) = 0.043
*S* = 1.05
3213 reflections
133 parameters
3 restraints
All H-atom parameters refined
Δρ_max_ = 0.20 e Å^−3^
Δρ_min_ = −0.13 e Å^−3^

### 

Data collection: *COLLECT* (Nonius, 1999 ▶); cell refinement: *PEAKREF* (Schreurs, 2008 ▶); data reduction: *Eval15* (Schreurs *et al.*, 2010 ▶) and *TWINABS-2008/4* (Sheldrick, 2008*a* ▶); program(s) used to solve structure: *SHELXS97* (Sheldrick, 2008*b* ▶); program(s) used to refine structure: *SHELXL97* (Sheldrick, 2008*b* ▶); molecular graphics: *PLATON* (Spek, 2009 ▶); software used to prepare material for publication: manual editing of *SHELXL* CIF file.

## Supplementary Material

Crystal structure: contains datablocks I, global. DOI: 10.1107/S160053681100420X/ez2229sup1.cif

Structure factors: contains datablocks I. DOI: 10.1107/S160053681100420X/ez2229Isup2.hkl

Additional supplementary materials:  crystallographic information; 3D view; checkCIF report

## Figures and Tables

**Table 1:** Hydrogen-bond geometry (Å, °)

*D*—H⋯*A*	*D*—H	H⋯*A*	*D*⋯*A*	*D*—H⋯*A*
N1—H1*N*⋯Cl1	0.909 (11)	2.418 (11)	3.3007 (7)	163.9 (9)
N1—H2*N*⋯Cl1^i^	0.895 (12)	2.275 (12)	3.1665 (6)	174.2 (10)
N1—H3*N*⋯O3	0.908 (12)	1.888 (12)	2.7519 (9)	158.2 (9)
O3—H1*O*⋯Cl1^ii^	0.860 (18)	2.364 (18)	3.2220 (7)	175.1 (15)
O3—H2*O*⋯Cl1^iii^	0.808 (17)	2.470 (17)	3.2613 (7)	166.9 (14)
C2—H2⋯O2^i^	0.901 (11)	2.385 (10)	3.1302 (9)	140.0 (8)

Symmetry codes: (i) 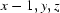; (ii) 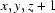; (iii) 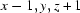.

**Fig. 1.**
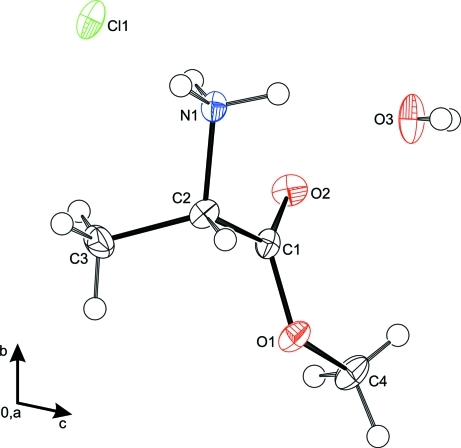
Molecular structure of (I), with atom labels and 50% probability displacement ellipsoids for non-H atoms.

**Fig. 2.**
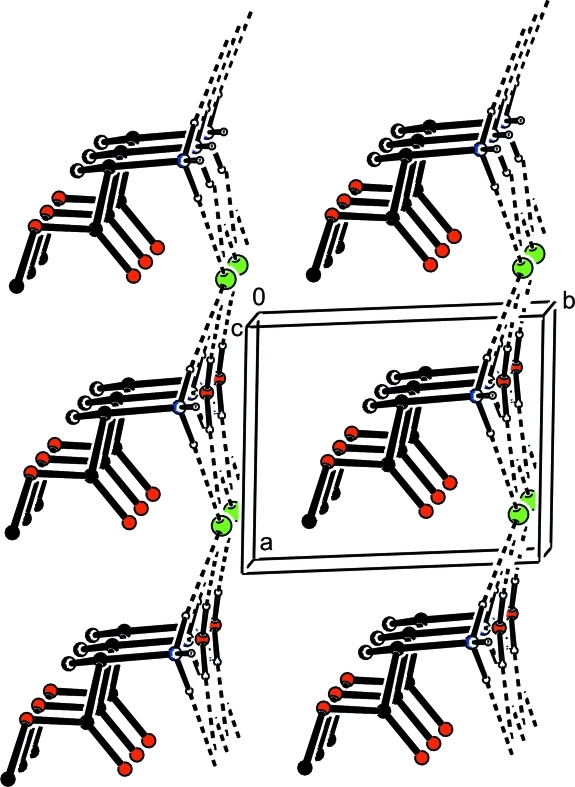
Formation of two-dimensional sheets parallel to (010) by hydrogen bonding in (I). Hydrogen bonds are drawn as dashed lines. H atoms not involved in hydrogen bonding have been omitted for clarity.

**Fig. 3.**
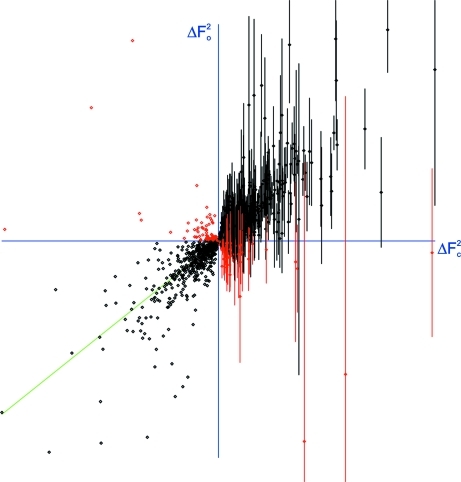
Bijvoet pairs in the non-overlapping reflections of the major twin component in (I). Scatter plot prepared by PLATON (Spek, 2009). 622 pairs are shown with Δobs > 0.25σ(Δobs). 534 Reflections confirming the absolute structure are drawn in black. 88 Reflections with the wrong sign are shown in red.

## supplementary crystallographic information

### Comment

In the context of our ongoing studies of absolute structure determinations of hydrochlorides of amino acid esters, we determined the structure of the title compound (I). The related L-serine methyl ester hydrochloride (Schouten & Lutz, 2009) has an extended backbone with O–C–C–N and C–O–C–C torsion angles of -175.99 (7) and 179.72 (7)°, respectively. In (I), the O–C–C–N torsion angle is 155.83 (6)° indicating a significant deviation from an extended backbone. The C–O–C–C torsion angle of 177.22 (6)° is again close to a *trans* conformation (Fig. 1).

Two H atoms of the ammonium moiety are involved in hydrogen bonds with chloride anions as acceptors. This results in a one-dimensional chain in the a-direction. These two hydrogen bonds have significantly different lengths: the N1···Cl1 distance is 3.3007 (7) Å, while the N1···Cl1 (*x* - 1, *y*, *z*) distance is 3.1665 (6) Å. The third ammonium H atom is hydrogen bonded to the co-crystallized lattice water molecule, which itself donates two hydrogen bonds to chlorides. The water thus links the one-dimensional chains into a two-dimensional network, which is parallel to the *a*,*c*-plane (Fig. 2). In the *b*-direction the hydrogen bonded layers of the ammonium moieties, chloride anions and lattice water molecules are alternating with the organic part of the alanine methyl ester. The O atoms of the ester functionality are not involved in strong intermolecular interactions, but there is a weak C—H···O bond with the ester O2 as acceptor (Table 1).

The crystal of (I) appeared to be twinned with a twofold rotation about *hkl*=(0,0,1) as twin operation. This twin relation was taken into account during the intensity integration with Eval15 (Schreurs *et al.*, 2010) and the refinement (Herbst-Irmer & Sheldrick, 2002). As can easily be verified in Fig. 2, the twinning operation results in reversed stacking of the two-dimensional hydrogen bonded networks. At the twinning boundaries the polar and ionic groups involved in the hydrogen bonds must approach each other in the direction of the *b* axis and the alternation of polar and apolar moieties is broken. These stacking faults might be accompanied by shifts of the layers for a better structural fit. Such dislocations often depend on the way the twin was generated (Cahn, 1954), which has not been investigated in the present study of (I). In the macroscopic shape of the crystal of (I), faces *hkl*=(0,0,1) and (0,0,1) have the smallest dimensions.

For the determination of the absolute structure, reflections with inverted indices were introduced into the dataset using the TWINABS software (Sheldrick, 2008*a*). Thus there were in total four twin domains included in the refinement. The corresponding twin fractions refined to 0.86 (2) and 0.104 (4) for the non-merohedral domains, and 0.03 (2) and 0.005 (4) for the corresponding inverted domains. The latter values are very close to zero and we can consider the enantiopurity as proven, but it should be noted that the two twin fractions of the inverted domains are in the least-squares refinement highly correlated with each other (correlation -0.999).

Because of this correlation we also performed a single-crystal refinement only on the non-overlapping reflections of the major twin domain. This dataset has a completeness of 82% (1447 unique reflections) and the coverage of Bijvoet pairs is 80% (712 pairs). Here, the Flack parameter (Flack, 1983) refined to a value of *x*=0.04 (3). On these data also an analysis according to Hooft *et al.* (2008) was performed. Assuming a Gaussian distribution of σ(I) the absolute structure parameter was calculated as *y*=0.044 (9). A plot of the Bijvoet differences is shown in Fig. 3.

### Experimental

Crystalline L-alanine methyl ester (Aldrich) was dissolved in technical ethanol. Evaporation at room temperature resulted in a viscous liquid. Crystallization was initiated by adding a seed crystal of the crystalline starting material.

### Refinement

The data set in HKLF-5 format (Herbst-Irmer & Sheldrick, 2002) contains non-overlapping reflections of both twin components, respectively, together with the overlapping reflections. Equivalent reflections were merged with TWINABS (Sheldrick, 2008*a*) prior to the least-squares refinement. The same software was used to introduce the inverted reflections for the absolute structure determination.

### Crystal data

**Table:**

C_4_H_10_NO_2_^+^·Cl^−^·H_2_O	*Z* = 1
*M**_r_* = 157.60	*F*(000) = 84
Triclinic, *P*1	*D*_x_ = 1.349 Mg m^−^^3^
Hall symbol: P 1	Mo *K*α radiation, λ = 0.71073 Å
*a* = 4.9461 (4) Å	Cell parameters from 5169 reflections
*b* = 6.0134 (4) Å	θ = 3.5–27.5°
*c* = 6.6853 (5) Å	µ = 0.44 mm^−^^1^
α = 101.833 (4)°	*T* = 110 K
β = 93.533 (3)°	Plate, colourless
γ = 92.112 (4)°	0.39 × 0.29 × 0.12 mm
*V* = 194.00 (2) Å^3^	

### Data collection

**Table:**

Nonius KappaCCD diffractometer	3213 independent reflections
Radiation source: rotating anode	3180 reflections with *I* > 2σ(*I*)
graphite	*R*_int_ = 0.019
φ and ω scans	θ_max_ = 27.7°, θ_min_ = 3.1°
Absorption correction: multi-scan (TWINABS-2008/4; Sheldrick, 2008*a*)	*h* = −6→6
*T*_min_ = 0.69, *T*_max_ = 0.75	*k* = −7→7
11388 measured reflections	*l* = −8→8

### Refinement

**Table:**

Refinement on *F*^2^	Primary atom site location: structure-invariant direct methods
Least-squares matrix: full	Secondary atom site location: difference Fourier map
*R*[*F*^2^ > 2σ(*F*^2^)] = 0.015	Hydrogen site location: difference Fourier map
*wR*(*F*^2^) = 0.043	All H-atom parameters refined
*S* = 1.05	*w* = 1/[σ^2^(*F*_o_^2^) + (0.0283*P*)^2^ + 0.0041*P*] where *P* = (*F*_o_^2^ + 2*F*_c_^2^)/3
3213 reflections	(Δ/σ)_max_ = 0.005
133 parameters	Δρ_max_ = 0.20 e Å^−^^3^
3 restraints	Δρ_min_ = −0.13 e Å^−^^3^

### Special details

**Table:**

Geometry. All e.s.d.'s (except the e.s.d. in the dihedral angle between two l.s. planes) are estimated using the full covariance matrix. The cell e.s.d.'s are taken into account individually in the estimation of e.s.d.'s in distances, angles and torsion angles; correlations between e.s.d.'s in cell parameters are only used when they are defined by crystal symmetry. An approximate (isotropic) treatment of cell e.s.d.'s is used for estimating e.s.d.'s involving l.s. planes.
Refinement. Refinement of *F*^2^ against ALL reflections. The weighted *R*-factor *wR* and goodness of fit *S* are based on *F*^2^, conventional *R*-factors *R* are based on *F*, with *F* set to zero for negative *F*^2^. The threshold expression of *F*^2^ > σ(*F*^2^) is used only for calculating *R*-factors(gt) *etc*. and is not relevant to the choice of reflections for refinement. *R*-factors based on *F*^2^ are statistically about twice as large as those based on *F*, and *R*- factors based on ALL data will be even larger.

### Fractional atomic coordinates and isotropic or equivalent isotropic displacement parameters (Å^2^)

**Table:**

	*x*	*y*	*z*	*U*_iso_*/*U*_eq_	
O1	0.52552 (12)	0.30665 (9)	0.60201 (9)	0.01877 (12)	
O2	0.74232 (12)	0.64013 (9)	0.59202 (9)	0.01863 (12)	
N1	0.29086 (13)	0.79507 (11)	0.43187 (9)	0.01456 (12)	
H1N	0.447 (2)	0.8444 (16)	0.3859 (16)	0.018 (2)*	
H2N	0.143 (2)	0.8271 (19)	0.3614 (17)	0.021 (3)*	
H3N	0.284 (2)	0.8560 (18)	0.5673 (18)	0.021 (2)*	
C1	0.54988 (14)	0.50654 (11)	0.54561 (10)	0.01343 (14)	
C2	0.30541 (15)	0.54425 (12)	0.41118 (12)	0.01473 (14)	
H2	0.150 (2)	0.4952 (16)	0.4556 (15)	0.016 (2)*	
C3	0.3288 (2)	0.42892 (16)	0.18812 (15)	0.02366 (18)	
H3A	0.171 (3)	0.452 (2)	0.110 (2)	0.047 (4)*	
H3B	0.494 (4)	0.489 (3)	0.146 (3)	0.060 (5)*	
H3C	0.331 (2)	0.273 (2)	0.1796 (19)	0.031 (3)*	
C4	0.75776 (19)	0.25176 (15)	0.72382 (14)	0.02231 (18)	
H4A	0.909 (3)	0.225 (2)	0.644 (2)	0.040 (3)*	
H4B	0.695 (4)	0.118 (3)	0.779 (3)	0.064 (4)*	
H4C	0.819 (4)	0.360 (3)	0.821 (3)	0.052 (4)*	
Cl1	0.793162 (15)	0.934964 (15)	0.171315 (15)	0.01891 (5)	
O3	0.26844 (13)	0.86392 (12)	0.85023 (9)	0.02491 (13)	
H1O	0.415 (4)	0.880 (3)	0.930 (3)	0.057 (5)*	
H2O	0.133 (3)	0.878 (2)	0.912 (2)	0.044 (4)*	

### Atomic displacement parameters (Å^2^)

**Table:**

	*U*^11^	*U*^22^	*U*^33^	*U*^12^	*U*^13^	*U*^23^
O1	0.0167 (3)	0.0218 (3)	0.0201 (3)	−0.0007 (2)	−0.0021 (2)	0.0111 (2)
O2	0.0107 (3)	0.0205 (3)	0.0253 (3)	0.0016 (2)	−0.0013 (2)	0.0067 (2)
N1	0.0123 (3)	0.0188 (3)	0.0132 (3)	0.0052 (2)	0.0008 (2)	0.0038 (2)
C1	0.0107 (4)	0.0182 (3)	0.0123 (3)	0.0038 (3)	0.0042 (3)	0.0038 (3)
C2	0.0098 (3)	0.0180 (3)	0.0176 (3)	0.0008 (2)	0.0007 (3)	0.0066 (3)
C3	0.0296 (6)	0.0195 (3)	0.0186 (4)	0.0036 (3)	−0.0074 (4)	−0.0013 (3)
C4	0.0195 (4)	0.0277 (4)	0.0231 (4)	0.0026 (3)	−0.0034 (4)	0.0143 (3)
Cl1	0.01091 (8)	0.03258 (8)	0.01630 (8)	0.00378 (5)	0.00140 (5)	0.01156 (6)
O3	0.0129 (3)	0.0475 (4)	0.0132 (3)	0.0008 (3)	0.0011 (2)	0.0038 (2)

### Geometric parameters (Å, °)

**Table:**

O1—C1	1.3351 (8)	C2—H2	0.901 (11)
O1—C4	1.4541 (10)	C3—H3A	0.939 (14)
O2—C1	1.2052 (9)	C3—H3B	0.961 (18)
N1—C2	1.4909 (9)	C3—H3C	0.930 (12)
N1—H1N	0.909 (11)	C4—H4A	0.947 (14)
N1—H2N	0.895 (12)	C4—H4B	1.001 (17)
N1—H3N	0.908 (12)	C4—H4C	0.854 (18)
C1—C2	1.5137 (10)	O3—H1O	0.860 (18)
C2—C3	1.5236 (12)	O3—H2O	0.808 (17)
			
C1—O1—C4	115.00 (6)	C3—C2—H2	109.9 (6)
C2—N1—H1N	106.5 (6)	C2—C3—H3A	109.1 (9)
C2—N1—H2N	110.3 (7)	C2—C3—H3B	107.1 (10)
H1N—N1—H2N	112.5 (10)	H3A—C3—H3B	114.5 (13)
C2—N1—H3N	107.0 (7)	C2—C3—H3C	108.4 (8)
H1N—N1—H3N	110.0 (10)	H3A—C3—H3C	105.7 (11)
H2N—N1—H3N	110.2 (10)	H3B—C3—H3C	111.9 (12)
O2—C1—O1	125.01 (7)	O1—C4—H4A	110.8 (9)
O2—C1—C2	123.63 (6)	O1—C4—H4B	105.5 (10)
O1—C1—C2	111.36 (6)	H4A—C4—H4B	113.8 (13)
N1—C2—C1	106.75 (6)	O1—C4—H4C	114.3 (11)
N1—C2—C3	110.50 (6)	H4A—C4—H4C	102.3 (14)
C1—C2—C3	111.57 (6)	H4B—C4—H4C	110.4 (15)
N1—C2—H2	106.5 (6)	H1O—O3—H2O	112.9 (15)
C1—C2—H2	111.5 (6)		
			
C4—O1—C1—O2	−1.75 (11)	O1—C1—C2—N1	155.83 (6)
C4—O1—C1—C2	177.22 (6)	O2—C1—C2—C3	95.64 (9)
O2—C1—C2—N1	−25.18 (9)	O1—C1—C2—C3	−83.35 (8)

### Hydrogen-bond geometry (Å, °)

**Table:**

*D*—H···*A*	*D*—H	H···*A*	*D*···*A*	*D*—H···*A*
N1—H1N···Cl1	0.909 (11)	2.418 (11)	3.3007 (7)	163.9 (9)
N1—H2N···Cl1^i^	0.895 (12)	2.275 (12)	3.1665 (6)	174.2 (10)
N1—H3N···O3	0.908 (12)	1.888 (12)	2.7519 (9)	158.2 (9)
O3—H1O···Cl1^ii^	0.860 (18)	2.364 (18)	3.2220 (7)	175.1 (15)
O3—H2O···Cl1^iii^	0.808 (17)	2.470 (17)	3.2613 (7)	166.9 (14)
C2—H2···O2^i^	0.901 (11)	2.385 (10)	3.1302 (9)	140.0 (8)

Symmetry codes: (i) *x*−1, *y*, *z*; (ii) *x*, *y*, *z*+1; (iii) *x*−1, *y*, *z*+1.
